# Tunable magnetization steps in mixed valent ferromagnet Eu_2_CoMnO_6_

**DOI:** 10.1038/s41598-021-88950-w

**Published:** 2021-04-30

**Authors:** Nara Lee, Jong Hyuk Kim, Dong Gun Oh, Hyun Jun Shin, Hwan Young Choi, Sungkyun Choi, Younjung Jo, Young Jai Choi

**Affiliations:** 1 Department of Physics, Yonsei University, Seoul, 03722 Korea; 2 Center for Integrated Nanostructure Physics, Institute for Basic Science (IBS), Suwon, 16419 Republic of Korea; 3 Sungkyunkwan University (SKKU), Suwon, 16419 Republic of Korea; 4 Department of Physics, Kyungpook National University, Daegu, 41566 Korea

**Keywords:** Physics, Condensed-matter physics, Ferromagnetism, Magnetic properties and materials

## Abstract

Magnetic properties can be manipulated to enhance certain functionalities by tuning different material processing parameters. Here, we present the controllable magnetization steps of hysteresis loops in double-perovskite single crystals of Eu_2_CoMnO_6_. Ferromagnetic order emerges below *T*_C_ ≈ 122 K along the crystallographic *c* axis. The difficulty in altering Co^2+^ and Mn^4+^ ions naturally induces additional antiferromagnetic clusters in this system. Annealing the crystals in different gas environments modifies the mixed magnetic state, and results in the retardation (after O_2_-annealing) and bifurcation (after Ar-annealing) of the magnetization steps of isothermal magnetization. This remarkable variation offers an efficient approach for improving the magnetic properties of double-perovskite oxides.

## Introduction

Magnetic oxides composed of metal cations and oxygen anions are extensively studied due to the abundance of the elements and stability of the compounds. In particular, there have been various attempts to manipulate magnetic characteristics to achieve advantageous properties or to enhance desirable functionalities by tuning parameters such as hydrostatic pressure, chemical doping, and strain^1–5^. Double-perovskite oxides, in which transition metal ions are alternatingly located in octahedral oxygen environments, have been broadly investigated because of their fascinating magnetic properties. These properties include exchange bias^6–8^, magnetocaloric effect^9–12^, and multiferroicity^13–17^. The emergent properties arise from the intricate magnetic interactions and antiphase boundaries/antisite disorders between the mixed-valence magnetic ions^18,19^. In the case that a magnetic rare-earth ion is included, the additional ordering of the rare-earth magnetic moment at a lower temperature generates a significant modification of the magnetic properties^20–22^.

In double-perovskite R_2_CoMnO_6_ (R = La, …, Lu) compounds, the majority of alternating Co^2+^ and Mn^4+^ ions leads to the long-range magnetic order emerging from the ferromagnetic Co^2+^ and Mn^4+^ superexchange interactions, while the magnetic transition temperature varies from 48 K for Lu_2_CoMnO_6_^15^ to 204 K for La_2_CoMnO_6_^23^ depending on the size of the rare-earth ions. However, the incomplete alteration of Co^2+^ and Mn^4+^ ions naturally results in additional antiferromagnetic clusters which correspond to anti-sites of ionic disorders and/or antiphase boundaries that lead to Co^2+^–Co^2+^ or Mn^4+^–Mn^4+^ pairs^24,25^. Another type of antiferromagnetic cluster involving the valence state of Co^3+^–Mn^3+^ can also be formed^26^. In Lu_2_CoMnO_6_, the magnetic frustration associated with the nearest-neighbor ferromagnetic and next-nearest-neighbor antiferromagnetic couplings gives rise to the up–up–down–down (↑↑↓↓) spin ordering^27^. This spin configuration has been known to produce ferroelectricity perpendicular to the *c*-axis^13,15^ as a result of the cooperative O^2−^ displacements through the symmetric exchange striction^28–30^. In Er_2_CoMnO_6_, the activation of the ferrimagnetic order between Er^3+^ and ferromagnetic Co^2+^/Mn^4+^ sublattices exhibits an inversion of the magnetic hysteresis loop^31^. Furthermore, the additional small portion of multiferroic phase which may result from the ↑↑↓↓ spin order was observed simultaneously with the ferrimagnetic phase^22^. In Gd_2_CoMnO_6_ and Tb_2_CoMnO_6_, the orders of large rare-earth magnetic moments of Gd^3+^ and Tb^3+^ at *T*_Gd_ = 21 K and *T*_Tb_ = 15 K, respectively, reveal the giant anisotropic magnetocaloric effects^9,10,32^. It is evident from the previous investigations that a detailed understanding of distinct magnetic phases and interactions is essential for examining functional properties in double perovskites.

The Eu_2_CoMnO_6_ (ECMO) crystallizes in a monoclinic structure with a *P*2_1_/*n* space group, in which Co^2+^ and Mn^4+^ ions are alternatingly located in corner-shared O^2−^ octahedral environments. Ferromagnetic order from dominant Co^2+^ and Mn^4+^ superexchange interactions arises at *T*_C_ ≈ 120 K. Magnetic properties appear to be susceptible to the growth temperatures and gas annealing conditions after the growth^33,34^. However, the previous studies were done only on polycrystalline specimens, in which the physical properties are averaged out over all spatial orientations, interrupting detailed characterization of intrinsic and anisotropic properties. To overcome this obstruction, we grew single crystals of ECMO using the flux method. In this work, we have confirmed that the ferromagnetic order in ECMO single crystals appears along the crystallographic *c* axis at *T*_C_ = 122 K. Since a small number of antiferromagnetic clusters are naturally involved in the major ferromagnetic phase in a double-perovskite^24,25^, annealing in different atmospheres results in modification of mixed magnetic states and drastic changes in the magnetic hysteresis loop. Our results establish that the atmospheric environments in post-annealing play an important role in modifying the magnetic properties in mixed-valent double-perovskite magnets.

## Results and discussion

**Figure 1:**
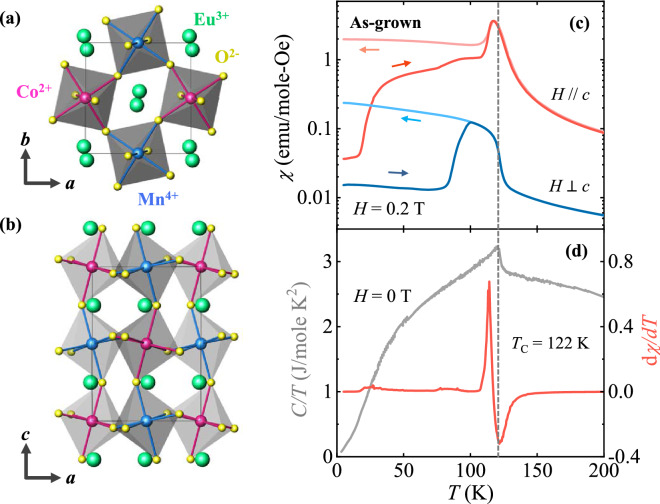
Crystallographic structure and temperature dependence of magnetic susceptibility in as-grown crystal. Views of the crystallographic structure of double-perovskite ECMO from the (**a**) *c* and (**b**) *b* axes. The green, pink, blue, and yellow spheres represent Eu^3+^, Co^2+^, Mn^4+^, and O^2−^ ions, respectively. (**c**) Temperature dependence of magnetic susceptibility, *χ* = *M/H*, shown in log scale, for the as-grown crystal in *H* = 0.2 T along and perpendicular to the crystallographic *c* axis and upon warming after ZFC and cooling in the same *H* (FC). The dotted line indicates the Curie temperature of *T*_C_ = 122 K. (**d**) Temperature derivative of ZFC *χ* along the *c* axis in *H* = 0.2 T and temperature dependence of specific heat divided by the temperature, *C/T*, measured in zero *H*.

The ECMO crystallizes in a monoclinic *P*2_1_/*n* structure with the lattice parameters, *a* = 5.3288(7) Å, *b* = 5.5824(7) Å, *c* = 7.5764(10) Å, and *β* = 89.9940(14)° (see Supplementary Information S1 for details). The structure of an ECMO crystal is depicted in Fig. 1a and b, viewed from the *c*- and *b*-axes, respectively. The O^2−^ octahedral cages are significantly distorted due to the relatively small radius of the Eu^3+^ ion. These series of compounds could be refined within two possible space groups, orthorhombic *Pbnm* (or *Pnma*) and monoclinic *P*2_1_/*n*^23^. However, the recent neutron diffraction studies on polycrystalline R_2_CoMnO_6_ (R = Y, Ho, and Tm) clearly demonstrate that the magnetic structure is accompanied by alternating Co^2+^ and Mn^4+^ spins^20,21^. The refinement result of the same magnetic moments for both Co and Mn ions suggests that valences of the ions are Co^2+^ and Mn^4+^ corresponding to high spin states (*S* = 3/2). The amount of antisite defects incorporated in such a compound was estimated as ~ 6%, which indicates that the physical properties with long-range ferromagnetic order can be interpeted within the frame of double-perovskite stucture, i.e., monoclinic *P*2_1_/*n* space group.

The magnetic properties of the as-grown ECMO crystal were investigated along and perpendicular to the *c* axis. Figure 1c shows the temperature (*T*) dependence of magnetic susceptibility described by magnetization divided by a magnetic field, *χ* = *M*/*H*, in log scale, measured upon warming in *H* = 0.2 T after zero-field cooling (ZFC) and upon cooling in the same *H* (FC). As the *T* decreases, the *χ* increases smoothly and the ferromagnetic order sets in at *T*_C_ = 122 K. The anomaly in the *T* dependence of heat capacity divided by *T* (*C*/*T*) in zero *H* and the trough in the *T* derivative of the ZFC *χ* curve for *H*||*c* also appear at *T*_C_ (Fig. 1d). A tiny magnitude of *χ* for *H*||*c* at 5 K after ZFC was observed due to an almost entirely demagnetized state. *χ* rises abruptly above ~ 17 K, which indicates thermally activated domain wall motion^35,36^. The *T* at which the ZFC and FC *χ* curves start to split were observed at 112 K for *H*||*c*, indicative of the onset of magnetic irreversibility. A sharp and positive peak of d*χ*/d*T* was observed at ~ 114 K, which represents an additional domain wall de-pinning process associated with the predominant long-range ferromagnetic order^37,38^. The *χ* values for the two different orientations exhibit strong magnetic anisotropy, which suggests that the spins are nearly aligned along the *c* axis, consistent with the neutron diffraction results that the ferromagnetic moments are aligned closed to the *c*-axis ^20,21^.

**Figure 2:**
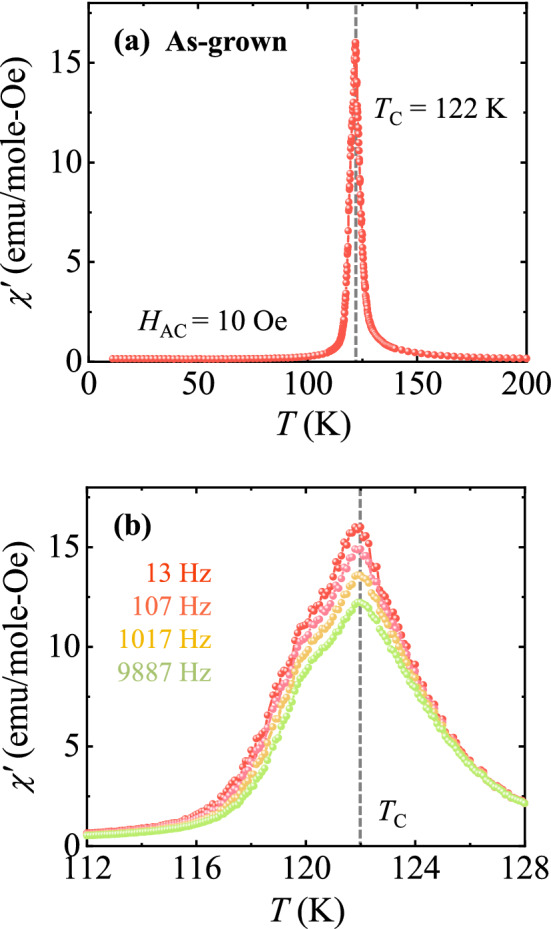
AC magnetic susceptibility for the as-grown crystal. Temperature dependence of (**a**) the real parts of AC magnetic susceptibility, *χ*′, measured at *f* = 13 Hz under zero DC magnetic-field and AC field excitation of 10 Oe and (**b**) *χ*′ at frequencies *f* = 13, 107, 1017, and 9887 Hz, near *T*_C_ (*T* = 112–128 K).

The *T* dependence of the AC magnetic *χ* was also measured for the as-grown crystal at the frequencies *f* = 13, 107, 1017, and 9887 Hz, under an AC excitation *H* of 10 Oe along the *c* axis (Fig. 2). In Fig. 2a, a peak appears at *T*_C_ at *f* = 13 Hz. As shown in Fig. 2b, the spread of AC *χ*, which occurs below *T*_C_, is manifestly ascribed to the additional domain wall motions. The peak height is reduced without any shift upon increasing *f*, which would be attributed to a magnetic disaccommodation process of pinned domain walls^20,39^. The absence of the peak shift in AC *χ* does not support that the magnetic irreversibility in DC *χ* (Fig. 1c) would be related to typical spin-glass behavior. In contrast, the recent AC *χ* measurements reported in polycrystalline specimens exhibit glassy behaviors^36,38^. This implies that Co and Mn ions would be better aligned in the single crystalline ECMO.

**Figure 3:**
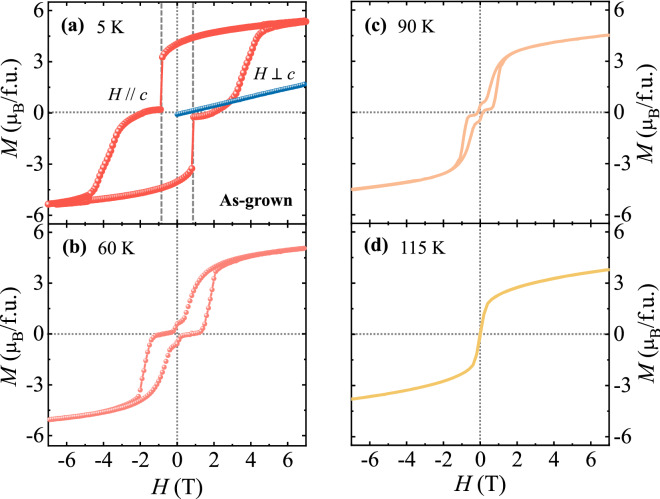
Isothermal magnetization for the as-grown crystal. Isothermal magnetization (**a**) along and perpendicular to the *c* axis measured at 5 K after cooling in *H* = 7 T and at (**b**) 60, (**c**) 90, and (**d**) 115 K, along the *c* axis measured after magnetic-field cooling in 7 T.

The *H* dependence of *M* at different temperatures for the as-grown crystal was examined. *M*(*H*) curves were obtained by sweeping *H* at 5, 60, 90, and 115 K after cooling the sample in *H* = 7 T. The highly anisotropic *M*(*H*) curves at 5 K are shown in Fig. 3a. The *M* in *H*||*c* at 7 T is found to be ~ 5.4 μ_B_/f.u._,_ smaller than the fully saturated value of 6.0 μ_B_/f.u., with the summation of Co^2+^ (*S* = 3/2) and Mn^4+^ (*S* = 3/2) states in a formula unit. This lack of magnetic saturation suggests the formation of anti-site disorders and antiphase boundaries, leading to antiferromagnetic Co^2+^–Co^2+^ or Mn^4+^–Mn^4+^ pairs^37,38,40^. The slight deviation of the ferromagnetic moment from the *c* axis would be another reason. In the case that misplaced magnetic ions are frustrated, the portion of anti-sites may increase further. Thus, the observed magnetic moments are compatible with the amount of antisite defects estimated as 6–8% from the neutron diffraction experiments on the isostructrual compounds^20,21^. To verify the oxygen content of the as-grown crystal, we used a thermogravimetric and differential thermal analysis under 5% H_2_/Ar atmosphere. The oxygen content was found to be 6.01 ± 0.019. In a recent X-ray photoemission spectroscopy experiment on a polycrystalline ECMO, the partial formation of Eu^2+^ moments (~ 5%) was observed^36^. The Eu^2+^ moments would be ordered antiferromagnetically to the Co^2+^/Mn^4+^ moments as observed in other members of the series^21,22^, which may act as one of the reasons for the reduced saturation *M*. The remnant *M* is attained as ~ 4.0 μ_B_/f.u., which demonstrates a squareness ratio of 0.74. The abrupt jumps of *M* in *H*||*c* occur at *H* =  ± 0.85 T, whereas the *M* in *H*⊥*c* increases linearly with a small magnitude. The change in magnitude of *M* from 3.27 to 0.17 μ_B_/f.u. at –0.85 T is caused by the alteration from the magnetic state with dominant up-spin domains to the almost demagnetized state. The knee-like feature of *M* above the large step in *H*||*c* would be influenced partly by the magnetic re-ordering or spin-flops of antiferromagnetic clusters. Upon increasing *T*, the magnetic hysteresis narrows and another *M* step near-zero *H* occurs as the remnant *M* value drops significantly (Fig. 3b,c). At 115 K, just below *T*_C_, the ferromagnetic behavior remains but the hysteresis and *M* steps vanish (Fig. 3d).

**Figure 4:**
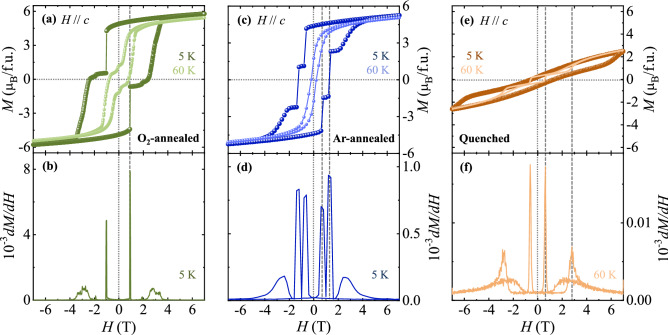
Isothermal magnetization for the annealed crystals. Isothermal magnetization for the (**a**) O_2_-annealed crystal, (**c**) Ar-annealed crystal, and (**e**) quenched crystal, along the *c* axis, up to 7 T, at *T* = 5 and 60 K, and the magnetic-field derivative of magnetization for the (**b**) O_2_-annealed crystal at 5 K, (**d**) Ar-annealed crystal 5 K, and (**f**) quenched crystal 60 K.

The different gas annealing conditions led to substantial modifications in the *H* dependence of *M*. In Fig. 4, the isothermal magnetizations along the *c* axis, taken at 5 K and 60 K, and the *H*-derivatives of magnetizations, are displayed for the O_2_-annealed crystal at 5 K (Fig. 4a,b, respectively), Ar-annealed crystal at 5 K (Fig. 4c,d, respectively) and quenched crystal at 60 K (Fig. 4e,f, respectively). After O_2_ gas annealing, the oxygen content of the O_2_-annealed crystal was estimated as 6.07 ± 0.008. The content of Eu^3+^ ions was also estimated to be 1.963 ± 0.008 from the EPMA mesurement, which may result from the partial replacements of Bi^3+^ ions inherent from Bi_2_O_3_ flux during the growth^33,34^. The *M* in *H*||*c* at 7 T is ~ 5.8 μ_B_/f.u. which is close to the saturated magnetic moment (Fig. 4a). In comparison with the as-grown crystal, the step of *M* at 5 K is slightly retarded, occurring at ± 0.91 T, which is manifestly displayed as a sharp peak in the derivative of *M* (Fig. 4b). The remnant *M* becomes larger, estimated as 4.87 μ_B_/f.u., and the squareness ratio is enhanced to 0.84. Additional small steps are found at ± 1.93 T, shown as broad bumps in the derivative of *M*. The *M* steps still remain at 60 K. The slow cooling procedure for the O_2_-annealed crystal improves the order of Co-Mn ion configuration. However, the excessive oxidation induces cationic vacancies that effectively pin the ferromagnetic domains^41^ despite the formation of a lower amount of anti-site defects. This conceivably explains the enhanced value and retarded step of *M*.

For the Ar-annealed crystal, isothermal *M* and its derivative at 5 K demonstrate two sharpened transitions occurring at 0.6 and 1.4 T with intermediate plateaus, as shown in Fig. 4c and d. The abrupt variations indicate the *H*-driven reversal from one saturated magnetic state to the other-direction saturated state through two-step *M* changes of magnetic domain walls. The *M* at 7 T and the remnant *M* are found to be 5.2 μ_B_/f.u. and 4.4 μ_B_/f.u., respectively, which determines a high squareness ratio of 0.85. More reduction of *M* at 7 T and oxygen content estimated as 5.87 ± 0.007 indicate that the oxygen-deficient atmosphere during Ar-anneling generates an additional portion of antiferromagnetic clusters. In more detail, the oxygen vacancies induce a reduced valence state such as the change from Mn^4+^ to Mn^3+^ to preserve the electroneutrality. This may lead to antiferromagnetic Co^2+^–Mn^3+^ pairs, giving an explanation for the reduced saturation value of *M*. Furthermore, the additional portion of antiferromagnetic pairs may break the coherence of ferromagnetic domains and lessen the magnetic inhomogeneity, which would present the two-step change of *M*. If the antisites of Mn^3+^–Mn^4+^ pairs are formed in the oxygen deficient condition, the double-exchange interactions between Mn^3+^ and Mn^4+^ moments are expected. However, these series of compounds are insulators, suggesting that the possible formation of Mn^3+^–Mn^4+^ pairs would be ruled out or the amount of Mn^3+^–Mn^4+^ pairs would be tiny and thus non-percolative in electrical conduction^23^. Furthermore, oxygen vacancies can contribute to the ionic disorders via the trapping of two Mn^3+^ ions. As a result, the traps in the vacant sites generate antiferromagnetic Mn^3+^–Mn^3+^ pairs^23^. A partial deficiency of Eu^3+^ ions was observed in N_2_ annealed polycrystalline ECMO^33,34^. In our single crystalline ECMO, the content of Eu^3+^ ions for the Ar annealed crystals was estimated as 1.931 ± 0.025 via the EPMA method. The presence of deficient Eu^3+^ ions may arise from the partial replacements of Bi^3+^ ions as well as oxygen deficient condition. The substantial alterations of shapes and steps in isothermal *M* curves suggest that Ar-annealing procedure engenders the reconstruction of antiferromagnetic clusters and modify the distribution of magnetic domain pinnings^42,43^. Unlike the O_2_-annealed case, these two sharp *M* steps completely disappear at 60 K, where only smooth ferromagnetic behavior is displayed. Both *M*(*H*) curves at 5 K for O^2−^ and Ar-annealed crystals exhibit slight linear slopes at the high *H* regime, resulting from the reorientation of a small portion of antiferromagnetic spins in antisites and/or antiphase boundaries^44^.

For the quenched crystal, the magnetic hysteresis loop at 5 K becomes narrow with the disappearance of sharp steps and includes the linear component in a broad *H* range (Fig. 4e). The hysteresis loop appears to be assymetric with a noticeable shift, which is ascribed to the minor hysteresis loop effect^45,46^. On the other hand, the *M* at 60 K reveals multiple steps at ± 0.6 and ± 2.75 T as shown in Fig. 4f. The quenching procedure may engender critical deterioration on crystal quality, and it thus destroys the square-shape response of *M*(*H*). The overall value of *M* is largely reduced and the *M* value at 7 T is found to be ~ 2.9 μ_B_/f.u., only about 50% of the fully saturated value, 6 μ_B_/f.u. The results suggest the prevailing formation of disorders and defects in which a considerable portion may contain additional antiferromagnetic clusters from antisites and/or antiphase boundaries. A close looking at the *M* process suggests the formation of mixed hard and soft ferromagnetic phases. As described for the isothermal *M* of ε-Fe_2_O_3_^47,48^, the inhomogeneous concentration of defects resulting in different pinned magnetic domains plays a crucial role in magnetically hysteretic behavior. High-defect regions would lead to a hard ferromagnetic behavior due to strongly pinned domains while few-defect regions would be relavant to a soft ferromagnetic behavior. Similar hysteretic behavior with a possible combination of hard and soft ferromagnetic phases was observed in the previous work on a polycrystalline Y_2_CoMnO_6_^20^.

**Figure 5:**
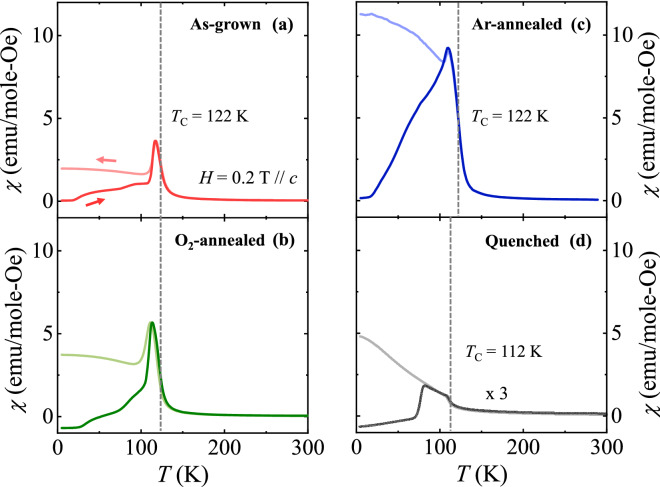
Magnetic susceptibility for the annealed crystals. Temperature dependence of ZFC and FC *χ* values displayed in linear scale along the *c* axis in *H* = 0.2 T for (**a**) the as-grown, (**b**) O_2_-annealed, (**c**) Ar-annealed, and (**d**) quenched crystals. The *χ* values are plotted after magnification by three times in scale for the quenched crystal.

The *T* dependence of *χ* is also influenced by the post-annealing atmosphere. The *T* dependence of ZFC and FC *χ* curves are displayed in linear scale for the as-grown, O_2_-annealed, Ar-annealed, and quenched crystals in Fig. 5. In O_2_-annealed and Ar-annealed crystals, *T*_C_ does not change, which implies that the gas-annealing procedure affects strongly on the *M* steps but not on the long-range ferromagnetic order^41^. For the O_2_-annealed crystal, a tiny negative magnitude of *χ* was observed after ZFC, at 5 K, due to a typical remanent *H* that remained negative upon cooling, as shown in Fig. 5b. As *T* is increased, the *χ* rises broadly with a shoulder-like feature, followed by a peak at ~ 115 K. The overall magnitude of *χ* after Ar-annealing increases, but the peak corresponding to the domain wall depinning process is found to be at the lower *T* ≈ 109 K. In the quenched crystal, the decrease of ferromagnetic transition temperature by 10 K (to *T*_C_ = 112 K) and the significant suppression of *χ* are observed due to the formation of additional defects and disorders. The weak glassy behavior in polycrystalline specimens indicates the more considerable formation of antiferromagnetic clusters. However, the averaging effect in physical properties of polycrystalline samples would disturb the observation of intrinsic and anisotropic properties. For example, magnetization steps in some of the polycrystalline ECMO appear to be less sharp than those of single crystals^24,33,34^ or to be completely vanished^33,34^. This suggests the importance of investigating single crystals in which intriguing physical properties based on strong magnetic anisotropy are made apparent.

Tunable *M* steps are achieved by reconstructing mixed magnetic states by annealing the crystals in different atmospheres. The step effect of the magnetic hysteresis loop has been theoretically investigated in intermixed ferromagnetic and antiferromagnetic states^49,50^. The mixture of ferromagnetic and antiferromagnetic interactions, combined with magnetic anisotropy and/or weak dipolar interaction, generates various shapes and steps in magnetic hysteresis loops depending on the relative ratio of two magnetic types. We do not yet have a microscopic understanding of the influence of annealing environments on the intriguing magnetic behaviors of ECMO. Thus, to reveal the mechanism for controllable *M* steps and to identify the wide spectrum of valences and exchange interactions of magnetic ions, further investigations of extensive magnetic properties for similar double-perovskite compounds are required.

## Conclusion

In summary, we have explored the magnetic properties of single-crystalline double-perovskite Eu_2_CoMnO_6_ prepared in different atmospheric annealing conditions. In the as-grown crystal, the magnetic susceptibility reveals ferromagnetic order along the *c* axis at *T*_C_ = 122 K, below which isothermal magnetization exhibits a step effect. The ferromagnetic and additional antiferromagnetic clusters are modified after annealing in different gas environments such that the temperature and magnetic-field dependencies of the magnetic properties vary markedly. We achieve the tuning of the magnetization steps in the as-grown crystal as retardation after O_2_-annealing and bifurcation after Ar-annealing. Our findings provide crucial clues for understanding the precise mechanism for alteration of mixed magnetic states and an efficient means to adjust the magnetic properties of double-perovskite compounds.

## Methods

We have synthesized rod-like single crystals of ECMO utilizing the conventional flux method with Bi_2_O_3_ flux in air^9,10,21^. The crystallographic structure of the EFO crystals was confirmed using an X-ray diffractometer (D/Max 2500, Rigaku Corp.). ECMO specimens in different atmospheric environments were prepared after the growth. The atmospheric environments were: an O_2_-annealing process (heated up to 1150 °C, held for 5 h, and cooled at the rate of 50 °C/h in O_2_ gas), Ar-annealed (heated up to 1150 °C, held for 5 h, and cooled at the rate of 50 °C/h in Ar gas), and quenched (heated for up to 1200 °C in air, held for 5 h, and quickly quenched down to room temperature). The oxygen contents were measured by a thermogravimetric and differential thermal analysis (TG–DTA; SDT Q600, TA instruments). Under 5% H_2_/Ar atmosphere, each sample was heated to 1000 °C with the rate of 5 °C/min. The Eu deficiency was measured using a Wavelength Dispersive X-ray Spectrometer in an EPMA (Electronic Probe Micro-Analyzer, JEOL JXA-8530F). The *T* and *H* dependences of DC magnetization were obtained at *T* = 5–300 K and *H* = – 7 to 7 T using a Magnetic Properties Measurement System (MPMS) and Physical Properties Measurement System (PPMS), manufactured by Quantum Design, Inc. The *T* dependence of AC magnetic susceptibility was measured using the PPMS. Specific heat was measured using the standard relaxation method in the PPMS.

## Supplementary Information

Supplementary Information
