# Supplementary material for: Tunable magnetization steps in mixed valent ferromagnet Eu2CoMnO6
Source: Sci Rep. 2021 Apr 30;11:9408. doi: 10.1038/s41598-021-88950-w (PMC8087831; doi:10.1038/s41598-021-88950-w)
Supplement: Supplementary file 1 — Supplementary Information [file 41598_2021_88950_MOESM1_ESM.pdf]

Supplementary information for **Tunable magnetization steps in mixed valent ferromagnet Eu_2CoMnO_6**

Nara Lee[1], Jong Hyuk Kim[1], Dong Gun Oh[1], Hyun Jun Shin[1], Hwan Young Choi[1], Sungkyun Choi[2,3], Younjung Jo[4], and Young Jai Choi[1]

[1]*Department of Physics, Yonsei University, Seoul 03722, Korea*
[2]*Center for Integrated Nanostructure Physics, Institute for Basic Science (IBS), Suwon 16419, Republic of Korea*
[3]*Sungkyunkwan University (SKKU), Suwon 16419, Republic of Korea*
[4]*Department of Physics, Kyungpook National University, Daegu 41566, Korea*

Correspondence and requests for materials should be addressed to Y. J. C. (phylove@yonsei.ac.kr).

S1. Characterization of crystallographic structures for as-grown and annealed Eu_2CoMnO_6 crystals

To identify the crystallographic structure of the as-grown Eu_2CoMnO_6 crystal, we performed high-resolution single crystal X-ray diffraction experiment at 223 K using a a Mo-source D8 Venture CCD X-ray diffractometer (Bruker Corp.). The crystal structure was refined with a JANA software[1]. Extinction was corrected using the isotropic Becker and Coppens model[2] implemented in the software. The structure was refined as monoclinic $P2_1/n$ space group (No. 14). The comparison between the calculated structure factor (F_{calc}) and the observed structure factor (F_{obs}) shows the well refined result. The details of structural parameters extracted are listed in Table S1. Reliability factors are goodness of fit (GOF or χ^2) = 3.19, R(obs) = 5.42 %, and wR(obs) = 8.20 %.

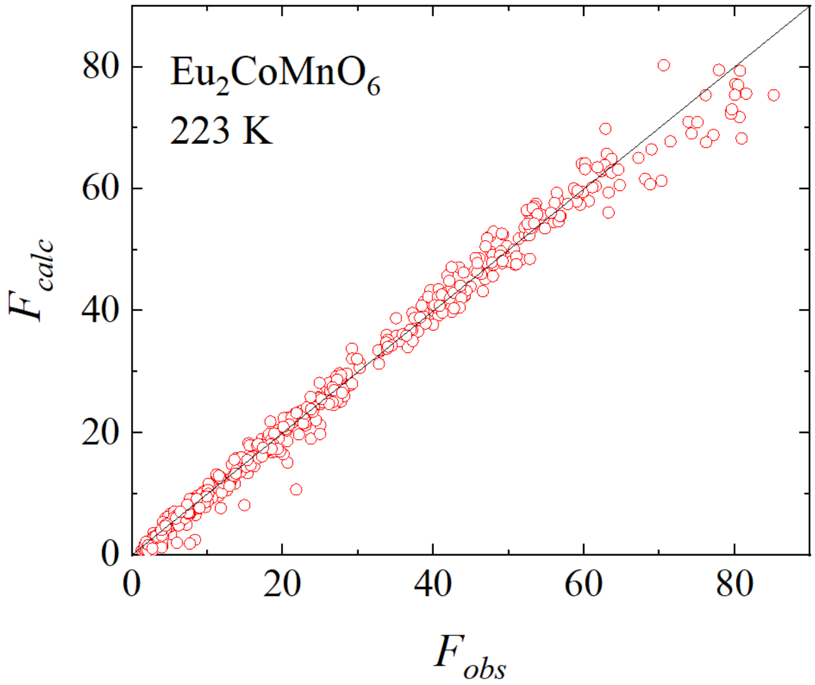

Figure S1. X-ray diffraction analysis presenting the calculated structure factor (F_{calc}) versus the observed structure factor (F_{obs}) using a unit cell with space group *P2₁/n*, corresponding to the crystal structure given in Table S1.

Table S1. Crystallographic data of Eu_2CoMnO_6 obtained from single-crystal x-ray diffraction ($P2_1/n$, a = 5.3288(7) Å, b = 5.5824(7) Å, c = 7.5764(10) Å, β = 89.9940(14)°, Z = 2). All sites are set to be fully occupied. U is the isotropic displacement factor (Å^2). The Mn ion's displacement factor, marked with an asterisk (*), is fixed as 0.001 to avoid a tiny negative value (~0.0015).

atom	site	*x*	*y*	*z*	*U* (Å^2)
Eu	4*e*	0.9864(1)	0.0611(1)	0.2500(1)	0.002(1)
Co	2*d*	0.5	0	0	0.006(1)
Mn	2*c*	0	0.5	0	0.001*
O1	4e	0.0938(18)	0.4753(14)	0.2483(9)	0.005(2)
O2	4e	0.6968(15)	0.3021(13)	0.4554(9)	0.008(2)
O3	4e	0.6919(15)	0.3000(12)	0.0496(9)	0.007(2)

We have also performed powder X-ray diffraction experiment for the as-grown and annealed samples at room temperature in a X-ray diffractometer (D/Max 2500, Rigaku Corp.) with Cu-

K_α radiation. The diffraction data were analyzed by the Rietveld refinement using the Fullprof software. The result suggests that Eu_2CoMnO_6 crystallizes in a monoclinic double perovskite ($P2_1/n$ space group). Further details of crystallographic data for the as-grown, Ar-annealed, and O_2-annealed crystals are summarized in Table S2. Note that there was difficulty in analyzing the X-ray diffraction data for the quenched crystal, probably due to the critical deterioration of crystal quality.

Table S2. Crystallographic data of Eu_2CoMnO_6 obtained from powder X-ray diffraction

	**As-grown**	**O_2-annealed**	**Ar-annealed**
structure	Monoclinic	Monoclinic	Monoclinic
space group	$P2_1/n$	$P2_1/n$	$P2_1/n$
a	5.34370(6)	5.34399(6)	5.33083(5)
b	5.59559(7)	5.59429(6)	5.57913(5)
c	7.60259(9)	7.60113(8)	7.57840(7)
β	89.996(9)	89.993(8)	89.95103(1)
Eu (x)	0.98828(2)	0.98695(19)	0.98551(7)
Eu (y)	0.06034(7)	0.06095(6)	0.06259(4)
Eu (z)	0.25276(5)	0.25175(9)	0.24650(1)
Co / Mn (x)	0.5/0	0.5/0	0.5/0
Co / Mn (y)	0/0.5	0/0.5	0/0.5
Co / Mn (z)	0/0	0/0	0/0
O1 (x)	0.08529(8)	0.09027(7)	0.89736(3)
O1 (y)	0.46885(6)	0.47173(6)	0.53330(3)
O1 (z)	0.22499(11)	0.24956(11)	0.74156(5)
O2 (x)	0.66775(3)	0.70532(12)	0.26162(3)
O2 (y)	0.30623(3)	0.30060(2)	0.19021(3)
O2 (z)	0.45482(2)	0.45604(12)	0.96400(5)
O3 (x)	0.71318(4)	0.70521(12)	0.65611(3)

O3 (y)	0.28574(3)	0.29772(2)	0.72020(3)
O3 (z)	0.05229(2)	0.04369(12)	0.56231(5)
R_p (x)	11.8	12.7	9.38
R_{wp} (y)	8.05	7.93	12.1
R_{exp} (z)	6.59	7.41	10.06
χ^2	1.49	1.15	1.44

References

[1] V. Petříček,M. Dušek, and L. Palatinus, Z. Kristallogr. 229, 345 (2014).

[2] P. J. Becker and P. Coppens, Acta Crystallogr., Sect. A: Found. Crystallogr. 30, 129 (1974); 30, 148 (1974); 31, 417 (1975).
